# In Search for Comparability: The PECUNIA Reference Unit Costs for Health and Social Care Services in Europe

**DOI:** 10.3390/ijerph19063500

**Published:** 2022-03-16

**Authors:** Susanne Mayer, Michael Berger, Alexander Konnopka, Valentin Brodszky, Silvia M. A. A. Evers, Leona Hakkaart-van Roijen, Mencia R. Guitérrez-Colosia, Luis Salvador-Carulla, A-La Park, William Hollingworth, Lidia García-Pérez, Judit Simon, on behalf of the PECUNIA Group

**Affiliations:** 1 Department of Health Economics, Center for Public Health, Medical University of Vienna, Kinderspitalgasse 15/1, 1090 Vienna, Austria; susanne.mayer@meduniwien.ac.at (S.M.); michael.a.berger@meduniwien.ac.at (M.B.); 2 Department of Health Economics and Health Services Research, University Medical Center Hamburg, 20246 Hamburg, Germany; a.konnopka@uke.de; 3 Department Psychology, MSH Medical School Hamburg, 20457 Hamburg, Germany; 4 Department of Health Economics, Institute of Economic and Public Policy, Corvinus University of Budapest, 1093 Budapest, Hungary; valentin.brodszky@uni-corvinus.hu; 5 Department of Health Services Research, Care and Public Health Research Institute (CAPHRI), Faculty of Health, Medicine and Life Sciences (FHML), Maastricht University, 6229 ER Maastricht, The Netherlands; s.evers@maastrichtuniversity.nl; 6 Centre of Economic Evaluation & Machine Learning, Trimbos Institute, 3521 VS Utrecht, The Netherlands; 7 Erasmus School of Health Policy & Management, Erasmus University Rotterdam, Burgemeester Oudlaan 50, 3062 PA Rotterdam, The Netherlands; hakkaart@eshpm.eur.nl; 8 Department of Psychology, Universidad Loyola Andalucía, 41704 Dos Hermanas, Spain; menciaruiz@uloyola.es; 9 Asociación Científica Psicost, 41704 Dos Hermanas, Spain; 10 Health Research Institute, Faculty of Health, University of Canberra, Canberra 2617, Australia; luis.salvador-carulla@canberra.edu.au; 11 Menzies Centre for Health Policy and Economics, School of Public Health, University of Sydney, Sydney 2006, Australia; 12 Care Policy and Evaluation Centre, Department of Health Policy, London School of Economics and Political Science, London WC2A 2AE, UK; a.park@lse.ac.uk; 13 Department of Population Health Sciences, Bristol Medical School, University of Bristol, 1-5 Whiteladies Rd, Bristol BS8 1NU, UK; william.hollingworth@bristol.ac.uk; 14 Servicio de Evaluación, Servicio Canario de la Salud (SESCS), Camino Candelaria Nº 44, 1ª Planta, El Rosario, 38109 Santa Cruz De Tenerife, Spain; lidia.garciaperez@sescs.es; 15 Department of Psychiatry, University of Oxford, Warneford Hospital, Oxford OX3 7JX, UK

**Keywords:** unit cost, reference cost, valuation, service, mental health, health care, social care, societal perspective, economic evaluation, PECUNIA

## Abstract

Improving the efficiency of mental healthcare service delivery by learning from international best-practice examples requires valid data, including robust unit costs, which currently often lack cross-country comparability. The European ProgrammE in Costing, resource use measurement and outcome valuation for Use in multi-sectoral National and International health economic evaluAtions (PECUNIA) aimed to harmonize the international unit cost development. This article presents the methodology and set of 36 externally validated, standardized reference unit costs (RUCs) for five health and social care services (general practitioner, dentist, help-line, day-care center, nursing home) in Austria, England, Germany, Hungary, The Netherlands, and Spain based on unambiguous service definitions using the extended DESDE PECUNIA coding framework. The resulting PECUNIA RUCs are largely comparable across countries, with any causes for deviations (e.g., country-specific scope of services) transparently documented. Even under standardized methods, notable limitations due to data-driven divergences in key costing parameters remain. Increased cross-country comparability by adopting a uniform methodology and definitions can advance the quality of evidence-based policy guidance derived from health economic evaluations. The PECUNIA RUCs are available free of charge and aim to significantly improve the quality and feasibility of future economic evaluations and their transferability across mental health systems.

## 1. Introduction

Mental health conditions have considerable economic impacts. Apart from the direct impacts in the microeconomic sphere of the mental health patients, they also extend to the macroeconomic level of national budgets. In the challenging environment of health care expenditure growing faster than the economy at large in many European countries [1], such fiscal aspects have important implications for the sustainable financing of healthcare systems. The circular character of this relationship aggravates this issue. The economic crisis exerts upward pressure on the incidence of mental health conditions in the population [2]. In Spain, for instance, where the economy and housing market were severely hit by the Great Recession from 2007 to 2009, evidence from primary care centers suggests that the frequency of mood, anxiety, somatoform, and alcohol-related disorders increased substantially in the aftermath of the Great Recession [3]. Greece, too, saw a rise in deaths from suicides as well as in the prevalence of mental health problems [4]. Adequate social security nets tend to mitigate such adverse health effects [5]. However, policymakers are typically under pressure to consolidate public budgets in the aftermath of economic crises, and the implementation of ill-devised austerity measures can further fuel the negative effects of economic crises on health [6,7]. This, in turn, has two relevant fiscal consequences: on the one hand, mental health conditions lower the individuals’ possibilities to participate in the labor force [8,9], reducing their productivity (sickness absence, presenteeism) or barring them from participating altogether. The fiscal consequence is the loss of important tax revenues. On the other hand, the individual’s need for mental healthcare services increases [10], which critically stretches the available resources in the healthcare system. The failure to provide mental healthcare services in time hence propels an unmet need for care in the population. The consequences are in turn felt throughout the entire economy.

Budget cuts in the health sector can reduce productivity and increase economic hardship in the population, leading to lower tax revenue and thereby creating pressure to consolidate budgets—a vicious circle whose consequences are not limited to the sphere of health and labor. An unmet need for mental health treatment and reduced tax revenue is also felt in other sectors of social life (e.g., criminal justice and education), exacerbating the problem at hand. To avoid this vicious circle, healthcare policymakers are well advised to base their policy action on international best-practice examples. For instance by tackling wasteful spending in high-cost healthcare services that do not yield benefits for patients (e.g., [11]), or by carefully adjusting policy measures to the characteristics of the national financing system to avoid misaligned incentives (e.g., soft budget constraints, [12]).

In this context, cross-country comparability of services, their costs, and outcomes is critical to deriving valid policy guidance based on international health economic evidence [13]. However, precisely this comparability is often lacking, for example, because definitions of healthcare services based on semantic equivalence are not sufficiently unambiguous (e.g., [14,15,16]) or because results are depending on the applied unit cost valuation methodology [17,18,19,20]. A recent application of six costing methods to the unit cost calculation of a general practitioner (GP) consultation following established methodologies in countries including the UK, The Netherlands, and Germany, revealed a staggering difference of 173% between the lowest and highest unit cost estimate [21]. Researchers have voiced the need for a higher level of harmonization in the form of a library of standardized country-level unit costs [19]. With regards to costing, such a tool also offers the potential for a sound foundation of cross-country validity for economic evaluations of healthcare interventions, thereby making it easier for policymakers to learn from international best-practice examples and estimate economic impacts of policy rollouts with increased precision and a society-wide perspective. Improved policy guidance helps to increase the efficiency and effectivity of healthcare policy, for example by avoiding the potentially catastrophic consequences of across-the-board spending cuts in healthcare budgets.

The aim of this article is to present the newly developed calculation methods for standardized reference unit costs (RUCs) in the PECUNIA (ProgrammE in Costing, resource use measurement and outcome valuation for Use in multi-sectoral National and International health economic evaluAtions) project, as well as the first RUC results for a core set of health and social care services in six European countries. Beyond outlining the foundation of the presented unit costs and relevant harmonization challenges, such methodological transparency in the development process may help future researchers to develop more comparable unit costs for additional services and in additional countries. In turn, this will allow policymakers to better inform their (mental) healthcare-related decisions, for instance by improving the validity of comparisons to best-practice examples in other countries, as well as the accuracy of cost estimates and value judgments for policy rollouts.

### The PECUNIA Project

A key motivation behind the Horizon 2020-funded European research project ProgrammE in Costing, resource use measurement and outcome valuation for Use in multi-sectoral National and International health economic evaluAtions (PECUNIA; 2018–2021) [22] was to establish internationally comparable RUCs. The PECUNIA project spanned six European countries (Austria, Germany, Hungary, The Netherlands, England/UK, and Spain) and was coordinated by the Medical University of Vienna. While some European countries have published freely accessible unit cost lists for health and social care services on the national level (e.g., The Netherlands [23,24,25,26], UK [27,28,29], Germany [30,31,32]), an up-to-date international, multi-country and multi-sectorial harmonized collection of reference unit cost estimates is currently not available. For example, the Health Benefits and Service Costs in Europe (HealthBasket) project (2004–2007) produced unit costs associated with the delivery of care for a total of ten inpatient and outpatient services in nine countries but concluded that a comparable methodology for this was not available [33]. Most recently, the European Healthcare and Social Cost Database (EU HCCD) established a set of available unit costs for nine countries in the healthcare sector, productivity losses, and informal care costs, but its focus was on the synthesis of existing unit costs rather than their comparability.

The activities in the PECUNIA project aiming at cross-country comparability were structured in four stages: (i) identification of services, (ii) definition of services, (iii) measurement, and (iv) valuation. While each stage built on the results of the preceding one, the stages measurement and valuation were largely developed parallel to each other. As the motivation for this article lies in the potential advances in the financing of (mental) health care, the discussion focuses on the results of the valuation stage. In each stage, the PECUNIA country-lead research teams in the six participating countries (five for development, one for validation) put emphasis on the comparability aspects.

Following the identification of core services and resources using the newly developed PECUNIA Care Atom concept, the DESDE (Description and Evaluation of Services and DirectoriEs) PECUNIA coding system—a further development of the DESDE Long Term Care operational coding system [34]—allowed the PECUNIA researchers to assign standardized, unambiguously defined codes for services with semantic interoperability by defining services based on their Main Type of Care (MTC) [34]. Thereby, PECUNIA offers a step towards solving an issue previously neglected in multi-national economic evaluations regarding the transferability of economic evidence internationally.

Another challenge relevant for national and international trial-based economic evaluations includes the lacking match between available unit costs and the units of activity measured in existing resource-use measurement (RUM) instruments (e.g., [35]), which was addressed in stage (iii) of the PECUNIA project. Although a wide variety of patient-reported RUM questionnaires are available for free use [36], the key advantage of the PECUNIA RUM instrument is its linkage to internationally comparable PECUNIA RUC estimates. The developed multi-sectoral, multi-country RUM instrument [37] is aligned with the PECUNIA costing tools and RUCs, which is an additional benefit of the PECUNIA toolbox [38].

Most important in the context of this article is the common methodological foundation in the valuation of the health and social care services. This foundation is achieved using the PECUNIA Reference Unit Cost calculation Templates (RUC Templates) that were developed, pilot-tested (in Austria, Germany, Hungary, The Netherlands, England/UK), and validated (in Spain). These templates [39,40,41] facilitate the standardized calculation of RUCs and follow a harmonized and transparent methodology [14] for more comparability of reference unit costs (RUCs) in different sectors affected by health care interventions (e.g., health and social care sectors, education sector, employment sector), and countries.

## 2. Materials and Methods

The piloted and validated PECUNIA RUC Templates for services [39,40,41] were used in the harmonized calculation of the PECUNIA RUCs, i.e., the monetary value of an average unit per service [42]. The Microsoft Excel (2013)-based unit cost calculation blueprints are designed for self-completion by researchers and allow for the adoption of a top-down micro-costing approach or a top-down gross-costing approach [20], depending on data availability or the specific cost objective of the unit cost calculation [43]. The templates may be completed with available secondary data, primary data specifically collected for unit costing purposes, or a combination of both data sources. To facilitate the primary data collection, complementary PECUNIA primary data collection templates are available. Accompanying PECUNIA RUC Aggregation/Weighting (RAW) data sheets support the calculation of an aggregated estimate from multiple service providers [40]. In line with the full cost recovery theory [42], all cost categories relevant for service provision (e.g., direct costs, overhead costs) are captured in the PECUNIA RUC Templates, or as a minimum, transparency in case any cost category is missing is given. The templates adopt a societal opportunity cost perspective in the unit cost calculation where applicable, differentiating between provider costs and out-of-pocket expenses per service to avoid double counting between different sectors, but capturing the overall unit cost independently of who pays. While primary and secondary data were the first-best choices in the RUC calculation, other data sources covering the full costs of the average service provision were considered as suitable alternative sources.

In line with the preceding PECUNIA stages, five core health and social care services [44,45] were identified as relevant resource use items for mental health conditions in the health and social care sector and selected for the RUC calculations in the six countries: GP (unit of measurement: per contact), dental care (per contact), nursing home (per night), health-related day-care center (per day), health-related help-line (per contact). These services were described in the DESDE PECUNIA coding system [34] to ensure comparability across countries and implemented in the PECUNIA RUM instrument [38]. It was left up to the PECUNIA country-lead research teams if specific mental health-related RUCs or general, non-mental health-specific RUCs were calculated (e.g., for day care center) depending on the relevance of the specific service in the given national health and social care system. With the new PECUNIA costing concept and methods, this distinction between mental-health and generic services can be clearly indicated [46].

The calculations were conducted in six countries (Austria, Germany, Hungary, The Netherlands, England/UK, and Spain), representing different types of healthcare systems, by the PECUNIA country-lead research teams. RUCs could be developed based on national-level secondary data (not older than ten years at the time of the calculation) or primary data collected directly from services providers.

RUCs were calculated in Euro for the year 2019 and when necessary adjusted to 2019 prices based on the national consumer price indices (CPI). RUCs in different currencies were converted to the Euro based on the Eurostat exchange rate [47]. As national-level unit costs are in general the starting point for both national and multi-national economic evaluations, the PECUNIA costing methods recommend the presentation of non-PPP (purchasing power parity) adjusted RUCs. Adjustments for PPP differences were made only to check the sensibility of the results, as a 2017 comparison of hospital prices against the OECD average (index = 100) revealed a considerable difference, e.g., between the United Kingdom (71) and Hungary (21) [48].

In alignment with the PECUNIA RUM, RUCs were calculated for (1) state/social-insurance funded services, (2) privately funded services, and/or (3) as a (weighted), representative mixed estimate for state-/social-insurance funded and privately funded services to accommodate for the underlying differences in national health care systems. For each service, the researchers specified the relevant national prototype DESDE PECUNIA code(s) for each RUC, thereby enabling comparable service definitions across countries. Where required, additional ESCO/ISCO (European Skills/Competences, qualifications and Occupations/International Standard Classification of Occupations) codes [49,50] and ICHI (International Classification of Health Interventions) codes [51] were assigned to provide further details on the profession involved in the service provision, and on the exact intervention type, respectively.

The level of compliance with the PECUNIA costing standards in the RUC calculation was summarized in an index (‘PECUNIA costing approach: service: 1 = PECUNIA costing standards fulfilled; 2 = PECUNIA costing standards not fully fulfilled’). Full compliance with the PECUNIA costing standards implies that the unit cost estimate was representative on the national level and that a PECUNIA top-down micro-costing, top-down gross-costing, or mixed composite approach was applied, and that no major limitations on the data were reported. On the contrary, PECUNIA costing standards were deemed as not fully fulfilled if the RUCs were not representative on the national level, a second-best data source (e.g., tariffs) was applied in the unit cost development, or major data limitations were reported. Fees such as tariffs were considered as a second-best option as they may not reflect the economic cost of the service provision but rather a negotiated price [21,52].

The country-level RUC calculation phase concluded with a structured external validation phase of the developed estimates. The researchers critically appraised the quality of the RUC estimate based on criteria such as: (i) the comparison of the unit cost with comparable (existing) estimates, (ii) external expert feedback via interview, or (iii) feedback from the original (primary or secondary) data provider. The outcome of the external validation phase was documented and reported in a dedicated index (‘1 = Positive feedback; 2 = Caution/Caveat; 3 = Not available’).

Based on the index on the PECUNIA costing standards combined with the index on the external validation outcome, a summary index on the ‘Level of the certainty’ of the RUC was included (1 = High certainty, 2 = Medium certainty; 3 = Low certainty).

All RUCs were included in the Microsoft Excel^®^-based (2013) PECUNIA multi-sector, multi-country RUC Compendium [53], which contains all PECUNIA RUCs developed for the different PECUNIA sectors, including the health and social care sectors. The PECUNIA RUC Compendium comprehensively and transparently reports the relevant resource use item/service costing details.

## 3. Results

In the PECUNIA project, a total of 36 RUCs (in Euro for 2019) for the core set of five health and social care services were calculated either using the PECUNIA RUC Templates directly or applying the underlying harmonized methodological approach. The summary statistics on the main RUC characteristics are presented in Table 1 and a summary of the details of the individual RUC estimates is provided in Table 2.

Out of all 36 health and social care RUCs, 26 RUCs (72%) have been newly calculated using the PECUNIA methods, whereas 10 RUCs (28%) are derived from existing unit cost estimates based on comparable methods and validated using the PECUNIA methods. Twenty-five RUCs (69%) are based on calculations by PECUNIA country-lead research teams drawing on available secondary data, on specifically collected primary data (11%, *n* = 4), or a combination of secondary and primary data (3%, *n* = 1). The RUCs are either based on a top-down gross-costing approach (69%, *n* = 25), a top-down micro-costing approach (17%, *n* = 6), or a composite approach (6%, *n* = 2), while for 3 RUCs (8%) the exact costing approach is unknown. This is the case when calculations were based on reimbursement data, prices or tariffs assumed to cover the full costs of the average service provision, but no direct economic costing information was available. Overall, 72% (*n* = 26) of the 36 RUCs refer to the national level and 27% (*n* = 10) to the sub-national (regional or local) level. RUCs on the sub-national level were calculated based on data inputs from single providers, which do not deliver their services at a wider geographic level, and hence cannot be considered nationally representative. More than half of the RUCs are based on recent data/existing estimates between 2017 and 2019 (56%, *n* = 20), whereas 5 RUCs (14%) used data younger than the reference year (2019). The most commonly used mode of external validation was expert (83%, *n* = 30) and/or data provider feedback (19%, *n* = 7). For 6 RUCs (17%) that were re-calculated, no external evaluation was carried out.

**Figure 1:**
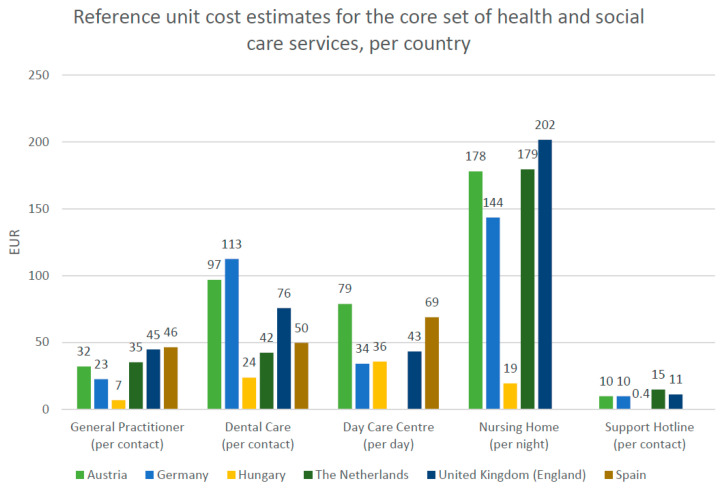
PECUNIA RUC estimates compatible with the PECUNIA RUM instrument per selected health and social care service for each PECUNIA partner country (in Euro, for year 2019; *n* = 27). Note: The figure presents the 27 RUCs that are suitable for valuation in combination with the PECUNIA RUM instrument.

Currently, 27 health and social care RUCs are suitable for direct matching with a PECUNIA RUM instrument item in terms of applicable national service funding and service scope. The remaining nine RUCs may be relevant for the valuation of a resource use item based on other RUM instruments or specific costing exercises (e.g., for specific dental care services and procedures in Hungary). The following presentation and discussion will focus on the 27 RUCs that are suitable for valuation in combination with the PECUNIA RUM instrument. Figure 1 illustrates these 27 RUCs in the six PECUNIA countries.

For GP services, RUCs per contact range between EUR 7 (Hungary) and EUR 46 (Spain). In this regard, it is important to note the potential biases in the RUC estimates for Spain (upward) as it was based on tariffs and Germany (downward) which does not cover add-on services due to missing data. Dental care service RUCs per contact range between EUR 24 (Hungary) and EUR 113 (Germany). The dental care service RUC estimate for The Netherlands refers to a dental check-up only in contrast to the RUCs in the other PECUNIA countries, which also take into account more time-intensive dental care services. An ICHI code [51] for a dental examination was therefore allocated to the Dutch RUC to make this deviation clearly visible. Further limitations to the dental care RUCs include the exclusion of patient co-payments (e.g., Hungary, Germany, England, Austria) and potential inaccuracy of the Hungarian estimate due to the substantial variation in costs depending on the specific procedure.

The RUC calculation for health-related day care centers and health-related support hotlines was complicated by the fact that in some countries such services predominantly target mental health patients, whereas in others all types of patients are covered. While the RUCs for health-related day care centers in Austria and Germany relate to all types of patients, the RUCs estimates for England, Hungary, and Spain refer exclusively to mental patients. Moreover, in contrast to the other countries, the RUC estimate for England captures direct costs only and does not include overheads. For (mental) health-related support lines, three RUCs (per contact) in Germany (EUR 10), England (EUR 11), and Hungary (EUR 0.4) refer to hotlines for health issues in general, while the RUCs in Austria (EUR 10) and The Netherlands (EUR 15) were based on support hotlines specialized in mental health. Lastly, RUCs for nursing homes (per night) vary between EUR 19 (Hungary) and EUR 202 (England).

For health-related day care centers in The Netherlands, as well as for health-related support hotlines and nursing homes in Spain, the PECUNIA country-lead research teams were not able to calculate the corresponding RUCs, as it was not possible to obtain the necessary primary or secondary data despite the researchers’ best efforts.

In total, 5 (18%) out of 28 RUCs that were subject to external validation had to be re-calculated based on the outcome of this step. Seventeen RUCs (63%) are rated with high certainty, 8 RUCs (30%) with medium certainty, and 2 RUCs (7%) are considered to be of low certainty. The latter concerns the RUC estimate for dental care services in Hungary, which is problematic due to the high variation in costs depending on the service type, and the RUC estimate for GP services in Spain, which is based on tariffs as a proxy and hence includes profit-margins.

## 4. Discussion

This article provides the first summary presentation of the PECUNIA RUC estimates for health and social care core services in six European countries calculated based on the harmonized PECUNIA service costing methods. These 36 developed RUCs for health and social care are included in the multi-sectoral, multi-country PECUNIA RUC Compendium, which is available free-of-charge following registration for non-commercial purposes [53]. The level of methodological transparency disclosed as standard in the compendium aims to support the development of more harmonized, comparable unit costs for additional services and countries in the future.

Although the work of the PECUNIA project showed that cross-country validity of cost estimates can be improved by harmonizing and transparently documenting costing methodology and service definitions, it also revealed that this does not suffice to resolve all comparability issues. Overall, Spain and England showed the highest level of RUCs based on the average country-rank for all healthcare services in the sample, with the important caveat that (1) there are fewer Spanish RUCs, (2) all but one referred to the regional level, and (3) one estimate has a potential upward bias as it was based on tariffs. In contrast, the RUCs were lowest in Hungary and Germany. Although the RUCs reflect differences in the wage and price levels (e.g., the markedly lower wage-level in Hungary), the ranking is robust to adjustments for PPP for actual individual consumption [54]. One crucial shortcoming of using a ranking is that it does not reflect the absolute size of the difference between the country-level RUCs, which may be rather small. The relative difference (in %) between the highest and the lowest country-level RUC is substantial, ranging from 231% (day care centers) to a staggering 3724% (support hotlines), largely driven by the overall lower RUCs in Hungary. Excluding Hungary from the sample, the difference becomes smaller but is still substantial ranging from 140% (nursing homes) to 265% (dental care). The large span between the highest and lowest RUC signals considerable heterogeneity in the costs of healthcare services even in an arguably homogeneous set of countries in terms of national income. The quality of the data remains a crucial heterogeneity component. Finally, the current PECUNIA RUC estimates were based on averages. While these are valid estimates, average unit costs have the potential drawback that they not reflective of individual services and may not be suitable in settings requiring detailed costs accounting for care intensity. For example, in the context of nursing home services, the actual cost depends very much on the intensity of the care required by individual residents. National payment systems hence often differentiate between levels of care intensity, but these definitions are not comparable across countries.

Several challenges were encountered in the RUC development phase resulting in potential limitations of the calculated RUCs. The COVID-19 pandemic affected the possibility of data collection. Although a comprehensive primary data collection strategy was originally foreseen, this was not ultimately not feasible. In an ideal scenario, a minimum number of 30 providers per country would be included in the aggregated RUC estimate, but for the presented RUC calculations, no minimum was set. However, the calculation of RUCs based on available secondary data resulted in nationally representative estimates and proved to be less resource intensive [39]. Around one-third of the RUCs were derived from existing estimates. These unit costs mainly refer to services provided in the UK and The Netherlands with underlying top-down micro-costing or gross-costing approaches. Both countries have established unit cost programs with largely comparable methodology as recently illustrated in the example of a GP consultation unit cost study in Austria [21]. Overall, for five services in two countries, a composite approach of mixing secondary data with tariffs was the only feasible option, with the external validation consequently yielding mixed results. In the context of working with secondary data, the external validation of the calculated RUCs, for example with data source providers (e.g., national statistics offices) proved to be a valuable step.

Since in the PECUNIA RUC Compendium all RUC input parameters are transparently reported, the included traffic light index allows future users of the PECUNIA RUC Compendium to assess the certainty and quality of the listed RUC estimates based on a simple classification. The RUCs with medium and low certainty may serve as a starting point for future improvements of these unit costs. They also point out the lack of robust, good quality secondary data and the need for improved access to such information or to specifically collected primary data on a nationally representative level.

## 5. Conclusions

Robust, comparable unit costs are fundamental to the quality of evidence-based policymaking. The PECUNIA costing concept (care atom and coding system) and tools (PECUNIA RUC Templates, PECUNIA RUC Compendium, PECUNIA RUM instrument) are the first comprehensive methodological toolkit offering the possibility to calculate internationally and across sectors harmonized RUC estimates. Even though costing is just one issue in the transferability of economic evaluations, their use will help increase the validity and quality of cross-country comparisons of future health economic evaluations in this regard. Future plans include the extension to additional services and resource use items and widening the country coverage. Researchers considering using these methods are encouraged to actively include national and international research institutions, governmental or other national public institutions, health care providers, for-profit organizations (e.g., pharma), and non-profit organizations in the calculation process from an early stage. These stakeholders, too, would benefit from the timesaving associated with a readily available, high-quality library of RUCs for several countries fully matching the PECUNIA RUM instrument and based on unambiguous service definitions.

While harmonizing methods and definitions is an important step, it does not suffice on its own to ensure cross-country comparability. It will be necessary to take further actions to improve the data quality and access as well, for example, by broadening the access to administrative data from public entities for research.

## Figures and Tables

**Table 1:** Summary statistics on the main characteristics of the 36 PECUNIA reference unit cost (RUC) estimates for health and social care services.

Reference Unit Costs (RUC) Characteristics (*N* = 36)	*n* (% of *N*)
Unit cost type:	
PECUNIA RUC	26 (72%)
Existing unit cost estimate	10 (28%)
Data type:	
Primary data	4 (11%)
Secondary data	25 (69%)
Combined data	1 (3%)
Unknown/undisclosed	6 (17%)
Unit cost calculation approach:	
Top-down micro-costing approach	25 (69%)
Top-down gross-costing approach	6 (17%)
Composite	2 (6%)
Unknown	3 (8%)
Representativeness of unit cost estimate:	
National	26 (72%)
Regional	9 (25%)
Local	1 (3%)
Original year(s) of RUC:	
2015 or older	6 (17%)
2016	2 (6%)
2017	3 (8%)
2018	2 (6%)
2019	15 (42%)
2020	1 (3%)
2021	4 (11%)
Multiple years	3 (8%)
External validation process: ^a^	
Comparison to existing unit cost estimate	4 (11%)
Expert feedback	30 (83%)
Data provider feedback	7 (19%)
No external evaluation	6 (17%)
Direct match with PECUNIA RUM items:	
Matching	27 (75%)
Not matching	9 (25%)

^a^ Note that some RUCs have been externally validated using more than one approach.

**Table 2:** Summary of the 36 PECUNIA Reference Unit Cost (RUC) estimates for health and social care services (in EURO, for year 2019).

No.	Country	Resource (Use) Item ^1^	Funding Source	Unit of Measurement	DESDE PECUNIA Code	Reference Unit Cost (EUR, 2019)	Representa-tiveness of RUC	Compatibility with PECUNIA RUM Instrument	Unit Cost Calculation Approach	Overall Certainty of RUC
01	Austria	Dental care	State/social insurance-funded	Per contact	SH-NX [K00-K14] O8.1	94.99	National	✘	Top-down gross-costing	2 (medium)
02	Austria	Dental care	Privately funded	Per contact	SH-NX [K00-K14] O8.1	120.00	National	✘	Unknown	2 (medium)
03	Austria	Dental care	Representative average	Per contact	SH-NX [K00-K14] O8.1	96.92	National	✔	Composite	2 (medium)
04	Austria	General practitioner	State/social insurance-funded	Per contact	SH-NX [ICD-10] O8.1	31.80	National	✘	Top-down micro-costing	1 (high)
05	Austria	General practitioner	Privately funded	Per contact	SH-NX [ICD-10] O8.1	45.45	National	✘	Unknown	2 (medium)
06	Austria	General practitioner	Representative average	Per contact	SH-NX [ICD-10] O8.1	32.04	National	✔	Composite	1 (high)
07	Austria	Health-related day care centre	State/social insurance-funded	Per day	SH-NX [ICD-10] D4.1; SH-NX [F00-F99] D4.1; SS-NX [ICF] D4.1	78.93	National	✔	Top-down gross-costing	2 (medium)
08	Austria	Health-related support line, mental health	Other	Per contact	SH-NX [F00-F99] I1.2.4e	9.98	National	✔	Top-down gross-costing	1 (high)
09	Austria	Nursing home	Representative average	Per night	SS-OX-R11; SH-AO [F00-F99] R11; SH-AO [ICF] R11	177.82	National	✔	Top-down gross-costing	1 (high)
10	Germany	Dental care	Representative average	Per contact	SH-NX [K00-K14] O8.1	112.53	National	✔	Top-down gross-costing	1 (high)
11	Germany	General practitioner	Representative average	Per contact	SH-NX [ICD-10] O8.1	22.68	National	✔	Top-down gross-costing	2 (medium)
12	Germany	Health-related day care centre	State/social insurance-funded	Per day	SH-NX [F00-F99] D4.1; SS-NX [ICF] D4.1	34.16	National	✔	Top-down gross-costing	2 (medium)
13	Germany	Health-related support line	State/social insurance-funded	Per contact	SH-NX [ICD-10] I1.2.4e	9.92	National	✔	Top-down gross-costing	1 (high)
14	Germany	Nursing home	State/social insurance-funded	Per night	SS-OX-R11; SH-AO [F00-F99] R11; SH-AO [ICF] R11	143.61	National	✔	Top-down gross-costing	2 (medium)
15	Spain	General practitioner	State/social insurance-funded	Per contact	SH-NX [ICD-10] O8.1	20.44	Regional	✘	Top-down gross-costing	2 (medium)
16	Spain	General practitioner	State/social insurance-funded	Per contact	SH-NX [ICD-10] O8.1	46.43	National	✔	Unknown	3 (low)
17	Spain	Dental care (at primary care centre)	State/social insurance-funded	Per contact	NX [ICD-10] O8.1-O10.1	49.68	Regional	✔	Top-down gross-costing	2 (medium)
18	Spain	Health-related day care centre, mental health	State/social insurance-funded	Per day	SH AX [F0-F9] D4.1	69.00	Regional	✔	Top-down gross-costing	2 (medium)
19	Hungary	Dental care	State/social insurance-funded	Per contact	SH-NX [K00-K14] O8.1	23.79	Regional	✔	Top-down gross-costing	3 (low)
20	Hungary	Dental care—checkout	Privately funded	Per contact	SH-NX [K00-K14] O8.1u + 2261.1 (ESCO)	18.19	Regional	✘	Top-down gross-costing	3 (low)
21	Hungary	Dental care—extraction	Privately funded	Per contact	SH-NX [K00-K14] O8.1u	40.96	Regional	✘	Top-down gross-costing	3 (low)
22	Hungary	Dental care—filling	Privately funded	Per contact	SH-NX [K00-K14] O8.1u	49.35	Regional	✘	Top-down gross-costing	3 (low)
23	Hungary	Dental care—small surgery	Privately funded	Per contact	SH-NX [K00-K14] O8.1u	78.24	Regional	✘	Top-down gross-costing	3 (low)
24	Hungary	General practitioner	State/social insurance-funded	Per contact	SH-NX [ICD-10] O8.1	6.82	Regional	✔	Top-down gross-costing	2 (medium)
25	Hungary	Health-related day care centre, mental health	State/social insurance-funded	Per day	SH-NX [ICD-10] D4.1; SH-NX [F00-F99] D4.1; SS-NX [ICF] D4.1	35.79	Local	✔	Top-down gross-costing	2 (medium)
26	Hungary	Health-related support line	Other	Per contact	SH-NX [ICD-10] I1.2.4e	0.40	National	✔	Top-down gross-costing	1 (high)
27	Hungary	Nursing home	State/social insurance-funded	Per night	SS-OX-R11; SH-AO [F00-F99] R11; SH-AO [ICF] R11	19.41	National	✔	Top-down gross-costing	2 (medium)
28	The Netherlands	Dental care	Representative average	Per contact	SH-NX [K00-K14] O8.1	42.40	National	✔	Top-down gross-costing	1 (high)
29	The Netherlands	General practitioner	State/social insurance-funded	Per contact	SH-NX [ICD-10] O8.1	35.24	National	✔	Top-down gross-costing	1 (high)
30	The Netherlands	Health-related support line, mental health	State/social insurance-funded	Per contact	SH-NX [F00-F99] I1.2.4e	14.89	National	✔	Top-down gross-costing	1 (high)
31	The Netherlands	Nursing home	Representative average	Per night	SS-OX-R11; SH-AO [F00-F99] R11; SH-AO [ICF] R11	179.43	National	✔	Top-down gross-costing	1 (high)
32	England	Dental care	Representative average	Per contact	SH-NX [K00-K14] O8.1	75.76	National	✔	Top-down micro-costing	1 (high)
33	England	General practitioner	State/social insurance-funded	Per contact	SH-NX [ICD-10] O8.1	44.69	National	✔	Top-down micro-costing	1 (high)
34	England	Health-related day care centre, mental health	State/social insurance-funded	Per day	SH-NX [F00-F99] D4.1; SS-NX [ICF] D4.1	43.29	National	✔	Top-down micro-costing	1 (high)
35	England	Health-related support line	State/social insurance-funded	Per contact	SH-NX [ICD-10]-I1.2.4e	11.19	National	✔	Top-down micro-costing	1 (high)
36	England	Nursing home	State/social insurance-funded	Per night	SS-OX-R11; SH-AO [F00-F99] R11; SH-AO [ICF] R11	201.65	National	✔	Top-down micro-costing	1 (high)

Note: ^1^ Resource use item definitions: Dental care: medical treatment and maintenance relating to the teeth; general practitioner: usually the first focal point for people with a health problem where basic care is provided and eventual referrals are coordinated; health-related day care centre: non-mental health-related: care or supervision provided during the day for ill persons by a voluntary organization/by a professional organization/by a social care facility, mental health-related: care or supervision provided during the day for mentally ill persons by a voluntary organization/by a professional organization/by a social care facility; health-related support line: a special phone and/or online service offering advice and support to people in distress.

## Data Availability

Data supporting reported results can be retrieved from: https://www.pecunia-project.eu/tools/ruc-compendium (accessed on 10 March 2022).
